# Serum antioxidant status and mortality from influenza and pneumonia in US adults

**DOI:** 10.1017/S1368980022000027

**Published:** 2022-01-10

**Authors:** Habyeong Kang, Howard Hu, Sung Kyun Park

**Affiliations:** 1 Department of Epidemiology, University of Michigan School of Public Health, Ann Arbor, MI 48109, USA; 2 Department of Preventive Medicine, Keck School of Medicine, University of Southern California, Los Angeles, CA, USA; 3 Department of Environmental Health Sciences, University of Michigan School of Public Health, Ann Arbor, MI, USA

**Keywords:** Micronutrients, Vitamin C, Lycopene, Oxidative stress, Infectious disease

## Abstract

**Objective:**

We examined the association between serum antioxidant status and mortality from influenza and pneumonia in US adults.

**Design:**

Serum concentrations of antioxidants included vitamin C, vitamin A, vitamin E, sum of *α*- and *β*-carotene, *β*-cryptoxanthin, lutein + zeaxanthin and lycopene. We computed total antioxidant capacity (TAC) as a measure of composite antioxidant status in serum. Survey-weighted Cox proportional hazard models were used to compute hazard ratios (HR) and 95 % CI comparing quartiles of each antioxidant and TAC.

**Setting:**

Data from the US National Health and Nutrition Examination Survey (NHANES)-III.

**Participants:**

A total of 7428 NHANES-III participants ≥45 years of age.

**Results:**

With a weighted-median follow-up of 16·8 years, 154 participants died from influenza/pneumonia. After adjustment for covariates, serum vitamin C, the sum of *α*- and *β*-carotene and TAC were nonlinearly associated with influenza/pneumonia mortality, with the statistically significant smallest HR at the third quartile *v*. the first quartile (HR = 0·38 (95 % CI: 0·19, 0·77), 0·29 (0·16, 0·51) and 0·30 (0·15, 0·59), respectively). HR comparing the fourth *v*. the first quartiles were weaker and nonsignificant: 0·57 (95 % CI: 0·27, 1·17), 0·70 (0·41, 1·19) and 0·65 (0·31, 1·35), respectively. Serum lycopene had a monotonic association with influenza/pneumonia mortality (HR = 0·43 (95 % CI: 0·23, 0·83) comparing the fourth *v*. the first quartile, *P*_for trend_ = 0·01).

**Conclusions:**

The current study suggests that antioxidant intake as reflected by serum concentrations may reduce mortality risk from influenza or pneumonia in the US general population. These findings warrant further confirmation in other populations with different settings (e.g. a shorter-term association with influenza infection).

Since the outbreak of coronavirus disease 2019 (COVID-19), there has been growing public interest in ‘immune-boosting’ by supplementation with micronutrients despite the skepticism of experts^(1)^. Nevertheless, given the dearth of cost-effective antiviral treatments apart from vaccination, supplementation of antioxidants (e.g. vitamins and carotenoids) has received growing attention as an inexpensive, widely available strategy for potentially reducing the morbidity (and, possibly, mortality) associated with COVID-19^(2,3)^.

Reactive oxygen species can accumulate in the process of viral infection, thereby leading to depletion of antioxidant capacity in the host cells^(4)^. Although reactive oxygen species may play a defensive role by killing the invading viruses, overwhelming production of reactive oxygen species caused by altered antioxidant status is detrimental to defending against the development and progress of infectious diseases. The excess oxidative stress can have a harmful effect on the host immune response by eliciting cytokine storm, a life-threatening exaggerated immune response^(5)^. Accumulated reactive oxygen species can also affect adaptive immunity by suppressing T cell responses^(6)^ and increase the formation of neutrophil extracellular traps, which leads to damage in lung epithelium and vascular endothelium^(5,6)^. In the process of viral replication, free radicals can induce mutation, by which the viruses may become more virulent^(7)^. These adverse effects of oxidative stress can be attenuated by treatment with antioxidants^(3,8)^.

There has been epidemiologic evidence of the protective effects of antioxidants on respiratory infections. A meta-analysis of randomised controlled trials (RCT) found that both prophylactic and therapeutic use of vitamin C had beneficial effects on the duration of the common cold^(9)^. A review concluded that prophylactic vitamin C might have protective effects against the common cold only in special situations such as under heavy physical activities or low dietary vitamin C intake^(10)^. This review also suggested that the evidence for the therapeutic effects of vitamin C is inconsistent^(10)^. In a study with children suffering from pneumonia, those who received 200 mg/d of vitamin C showed reduced severe pneumonia compared with the placebo group^(11)^. Although a meta-analysis and a review found several studies supporting the prophylactic or therapeutic role of vitamin C supplementation against pneumonia, the authors concluded that the evidence was not sufficient to be generalised to the ordinary population^(10,12)^. Vitamin E supplementation has also been shown to reduce the incidence of the common cold in RCT involving the elderly^(13,14)^. However, in general, epidemiologic evidence supporting the protective effects of antioxidants on influenza is limited, particularly in prospectively followed populations^(15)^.

In the current study, we investigated the prospective association of antioxidant status with influenza-related mortality using data from the US National Health and Nutrition Examination Survey (NHANES)-III database.

## Methods

### Study population

The current study used data from 9787 adult participants of NHANES-III aged 45 years and older that were collected between 1988 and 1994, linked with subsequent associated mortality data (NHANES-III Linked Mortality Public-Use File). We excluded participants who were missing data on mortality status (*n* 1203), serum antioxidants (*n* 586) or core covariates (*n* 570), yielding a final sample size of 7428 participants (online supplementary material, Supplemental Fig. 1).

### Serum antioxidants measurement

Among the micronutrients measured in the participants’ serum, we included vitamin C, vitamin A (as retinol), vitamin E (*α*-tocopherol) and the sum of *α*- and *β*-carotene, *β*-cryptoxanthin, lutein + zeaxanthin and lycopene. Serum concentrations of target antioxidants were measured by isocratic high-performance liquid chromatography. Analytical procedures for serum antioxidants were described in detail elsewhere^(16)^. Serum concentrations of the antioxidants were categorised into quartiles. Vitamin C, vitamin A and vitamin E were also categorised based on clinical recommendations on serum concentrations: low (<0·4 mg/dl), normal (0·4–<1·0 mg/dl) and saturated (≥1·0 mg/dl) for vitamin C^(17)^; deficient (<35·8 μg/dl), normal (35·8–85·9 μg/dl) and excess (>85·9 μg/dl) for vitamin A^(18)^ and deficient (<711 μg/dl), normal (711–1792 μg/dl) and excess (>1792 μg/dl) for vitamin E^(18)^.

To consider the composite effects of antioxidants, total antioxidant capacity (TAC) was calculated^(19)^. The TAC was originally proposed for dietary intake of antioxidants. The algorithm proposed by Floegel et al.^(19)^ was based on relative antioxidant capacity as vitamin C equivalent antioxidant capacity (VCEAC) determined by 2,2′-azino-bis(3-ethylbenzthiazoline-6-sulphonic acid) assay. Antioxidant capacity for each antioxidant was estimated by multiplying vitamin C equivalent antioxidant capacity and serum concentration of each antioxidant, and then serum TAC of each participant was calculated using the following equation:

$$\text{Serum TAC}=\sum [\text{serum antioxidant concentration}\left(\frac{100\;\text{g}}{\text{dl}}\right)\;\;\;\;\;\;\;\;\;\;\;\;\;\;\;\;\;\;\;\;\;\;\;\;\;\;\;\;\;\;\;\;\;\;\;\;\;\;\times \text{VCEAC of antioxidant}\left(\frac{\text{mgVCE}}{100\;\text{g}}\right)]$$

where VCE is vitamin C equivalent. The estimated serum TAC was also categorised into quartiles for data analysis.

### Mortality from influenza and pneumonia

NHANES-III data were linked with death certificate records from the National Death Index by the National Center for Health Statistics. Based on this linkage, it is available to identify mortality status, categorised leading cause of death and follow-up time from the NHANES-III interview date to death or end of follow-up (31 December 2015). We used the Public-Use Linked Mortality File for NHANES-III in this study. The categorised causes of deaths before 1999 were determined according to the ninth revision of the International Classification of Disease, Clinical Modification (ICD-9-CM) code^(20)^ while those from 1999 to 2015 were determined according to the tenth revision of the International Statistical Classification of Diseases and Related Health Problems (ICD-10) codes^(21)^. As the outcome of the current study, we considered mortality from ‘influenza and pneumonia’, which was categorised with ICD-10 codes J09-J18.

### Covariates

Demographic factors, socio-economic status and smoking history were collected by questionnaires. Age was top-coded at 90 years to protect the confidentiality of participants whose age was 90 years or older^(22)^. Since true ages of these participants are unknown, their ages were imputed with the weighted mean age of 93 years following the previous literature^(23)^. BMI was calculated by dividing weight (kg) by height squared (m^2^). Serum cholesterol was measured by an enzymatic method with a series of hydrolysis and oxidation reactions. Serum cotinine was measured by HPLC coupled with an atmospheric pressure chemical ionisation tandem MS.

Education and smoking history were selected as primary indicators of socio-economic status and exposure to tobacco smoking, respectively, because of fewer missing values in education and smoking history than other possible covariates (e.g. poverty:income ratio and serum cotinine). The residual confounding effects by poverty:income ratio and serum cotinine were evaluated in sensitivity analyses.

### Statistical analysis

All data and analytic code used in this study are available at https://github.com/um-mpeg/Antioxidant-Influenza-Pneumonia-Mortality. In all statistical analyses, the complex multistage sampling design was accounted for using the ‘survey’ package (version 4.0) in R (version 4.0.3; R Development Core Team). Survey-weighted Cox proportional hazard models were used to associate serum antioxidants with influenza/pneumonia mortality using the ‘svycoxph’ function in the survey package. Attained age, which is the age at influenza/pneumonia death in month, was used as the time scale based on a previous recommendation^(24)^. We computed hazard ratios (HR) and 95 % CI after adjustment for age, sex, race/ethnicity (non-Hispanic White, non-Hispanic Black, Mexican American and other) and NHANES III phase (1988–1991 and 1991–1994) as core covariates (Model 1) and additional adjustment for cholesterol level, BMI, smoking status (never, former and current), and education (< high school, high school diploma and > high school) (model 2). In addition, nonlinear relations between serum antioxidants and adjusted HR were visualised using survey-weighted restricted cubic spline (natural spline) models with three knots using the ‘ns’ function in the ‘splines’ package (version 4.0.3).

Adjusted cumulative hazard function plots for mortality from influenza/pneumonia were created stratified by serum antioxidant concentrations. If a clinical recommendation for the serum concentration of the antioxidant was available from previous literature, it was dichotomised by the recommendations. Otherwise, the cut-point for the dichotomisation was determined based on the HR estimation of the quartile group-based analyses. To control for confounding factors, we used a counterfactual approach^(25)^. Inverse probability weights were estimated for each individual by fitting logistic regression with the dichotomised antioxidants as the outcome and the covariates as predictors and then stabilised. By applying the stabilised inverse probability weights, we created cumulative hazard function plots, which are independent of confounders, using ‘ggsurvplot’ function in ‘survminer’ package (version 0.4.8).

Adjustment for chronic diseases, potential risk factors for influenza or pneumonia (i.e. hypertension, diabetes, heart attack, chronic lung diseases and cancer)^(26–28)^, was conducted as a sensitivity analysis (online supplementary material, Supplemental Table 3). Poverty:income ratio, serum cotinine (log-transformed) and supplement use were also additionally included in the sensitivity analyses to control for residual confounding by these variables. Alcohol consumption was also considered in a sensitivity analysis due to a substantial amount of missing values. Because antioxidants share common food sources, we evaluated possible confounding by adding another antioxidant as a covariate in the statistical models. Serum concentrations of antioxidants were measured only once at baseline. Therefore, another sensitivity analysis was performed after excluding very old adults aged ≥85 years and/or those with a follow-up ≥10 years given that the amount of antioxidants supplementation can change in very old age, and the antioxidant measurements of those with longer follow-up may not represent recent serum antioxidant status^(29,30)^. Data on 24 h recall-based dietary intake of the antioxidants were also available and tested as a sensitivity analysis (see details in Supplemental Methods).

## Results

Of the 7428 study participants, 154 participants died from influenza or pneumonia over the weighted median follow-up of 16·8 years (weighted mean incidence rate = 0·88 per 1000 person-years; Table 1). Among the quartile groups of serum TAC, the third quartile (Q3) had the lowest weighted mean mortality rate of 0·46 per 1000 person-years, while the weighted mean mortality rates of the first quartile (Q1) and the fourth quartile (Q4) were 1·21 and 1·22, respectively (Table 1). At baseline, the weighted mean age was 61·4 years. The weighted proportions of female and current or former smokers were 53·9 % and 57·9 %, respectively. The participants in Q4 of serum TAC were more likely to be older, female, non-Hispanic white and never smoker and to have lower BMI, higher total cholesterol and lower serum cotinine.

Table 2 shows the serum concentrations of the antioxidants and TAC at baseline. Vitamin E showed the highest median concentration of 1185 μg/dl followed by vitamin C (median concentration: 740 μg/dl). The median serum TAC was 1214 VCE/dl, and > 50 % of the serum TAC was contributed by the antioxidant capacity of serum vitamin C.

**Fig. 1:**
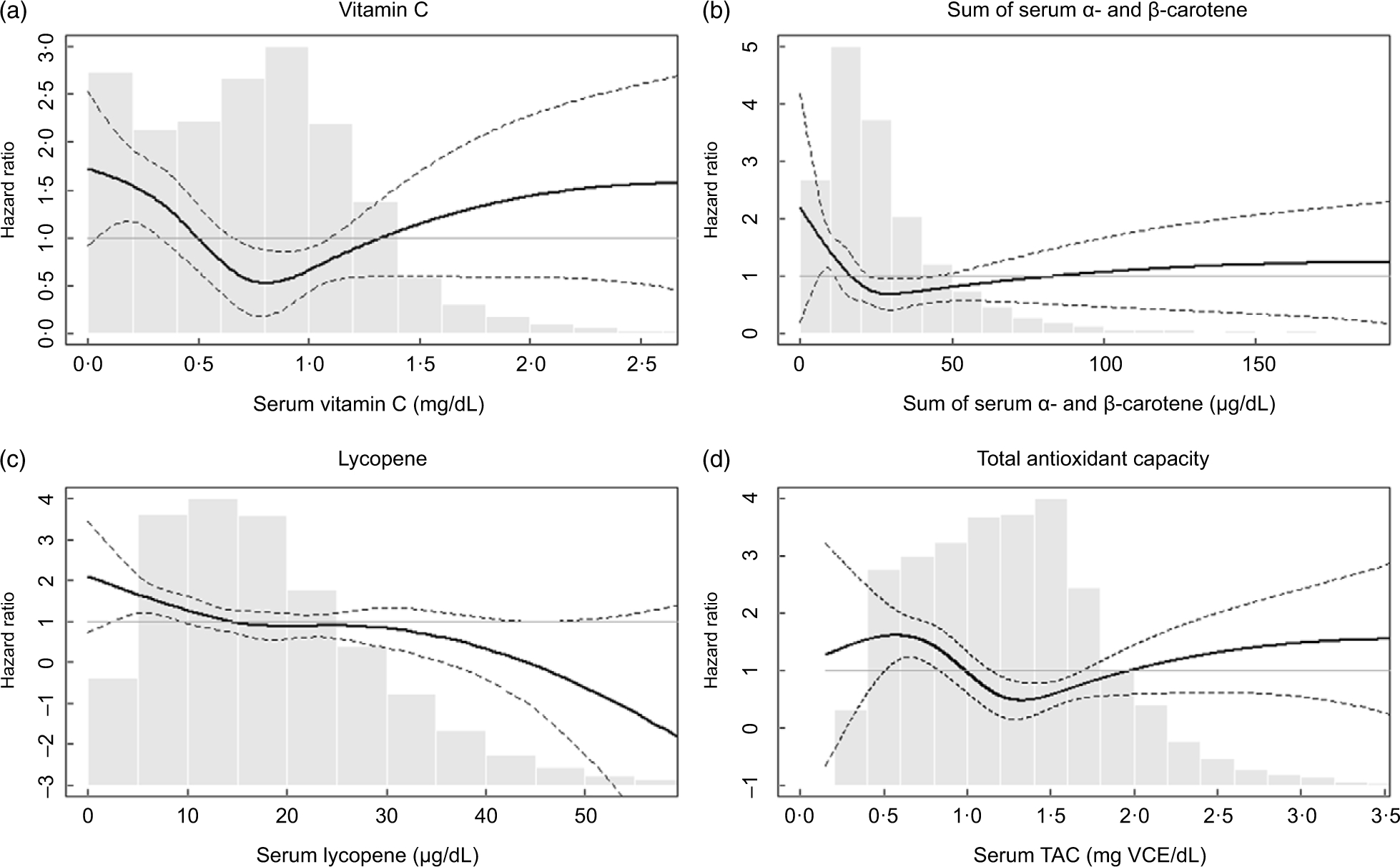
Adjusted hazard ratios (HR) for mortality from influenza/pneumonia by (a) serum vitamin C, (b) sum of *α*- and *β*-carotene, (c) lycopene and (d) total antioxidant capacity (TAC). The adjusted HR were presented as the bold lines based on survey-weighted restricted cubic spline models with three knots. The dotted lines indicate the upper and lower 95 % CI of the HR. The HR were adjusted for sex, race/ethnicity, NHANES III phase, education, cholesterol, BMI and smoking history. The grey bars represent histograms of the serum antioxidant levels or total antioxidant capacity

Table 3 shows HR for influenza/pneumonia mortality by quartiles of serum antioxidants and TAC at baseline. In model 1, which included sex, race/ethnicity and NHANES III phase, higher quartiles of several antioxidants were associated with lower HR compared with their lowest quartile. These associations remained similar after further adjustment for education, cholesterol, BMI and smoking status (model 2). Serum vitamin C, the sum of *α*- and *β*-carotene and TAC showed nonlinear associations with influenza/pneumonia mortality (HR (95 % CIs) for the third *v*. the first quartile: 0·38 (0·19, 0·77) for vitamin C, 0·29 (0·16, 0·51) for the sum of *α*- and *β*-carotene and 0·30 (0·15, 0·59) for TAC in model 2). The association with influenza/pneumonia mortality was also significant when serum vitamin C was categorised based on the clinical recommendation (online supplementary material, Supplemental Table 1). On the other hand, a monotonic association with influenza/pneumonia mortality was observed for serum lycopene (HR (95 % CI) for the fourth *v*. the first quartile: 0·43 (0·23, 0·83) in Model 2). No statistically significant associations were observed for other serum antioxidants. The association of serum lycopene with influenza/pneumonia mortality was monotonic, while U-shaped or L-shaped curves were observed for serum vitamin C, carotene and TAC (Fig. 1).

**Fig. 2:**
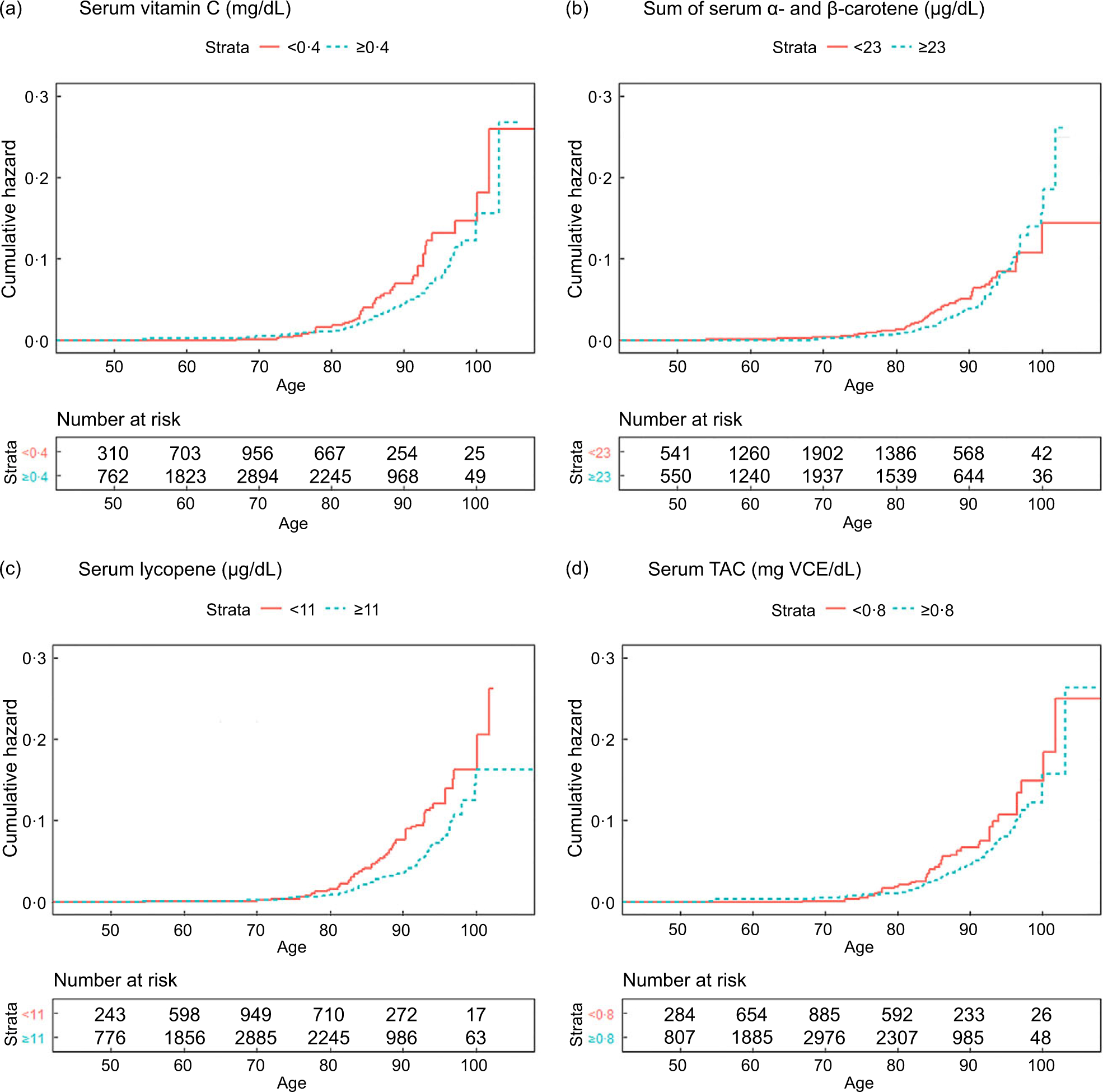
(colour online) Adjusted cumulative hazard plots for mortality from influenza/pneumonia (a) serum vitamin C, (b) sum of *α*- and *β*-carotene, (c) lycopene and (d) total antioxidant capacity (TAC). Serum vitamin C level was dichotomised by the clinical serum recommendation (0·4 mg/dl). Cutting points to dichotomise serum carotene, lycopene and TAC were determined based on hazard ratios (HR) estimated in the quartile analyses (23 μg/dl, 11 μg/dl and 0·8 mg VCE/dl, respectively). To account for confounding, inverse probability weights were estimated by fitting logistic regression with the dichotomised outcome (serum antioxidants or TAC) and predictors (age, sex, race/ethnicity, NHANES III phase, education, cholesterol, BMI and smoking history) and then stabilised. Adjusted cumulative hazard functions were fitted using the stabilised inverse probability weights

The adjusted cumulative hazard function plots showed that the cumulative hazard of mortality from influenza/pneumonia increased exponentially with age, and this increasing pattern was faster in the groups with lower serum concentrations than those with higher serum concentrations, except carotene (Fig. 2). The group with lower carotene level exhibited higher cumulative HR before around age 95 years, although it was reversed after that.

In sensitivity analyses, additional adjustment for either serum cotinine, poverty:income ratio, alcohol consumption, supplement use and status of several diseases did not change the HR (online supplementary material, Supplemental Tables 2 and 4). Moreover, the additional inclusion of either vitamin C or lycopene in the models did not substantially affect the HR (online supplementary material, Supplemental Table 5). When the population was restricted to those who were < 85 years old at baseline, the HR for higher serum levels of vitamin C, carotene, lycopene and TAC remained significant, while only carotene and lycopene had significant HR when follow-up time was restricted to < 10 years (online supplementary material, Supplemental Table 6). When we employed dietary antioxidants intake instead of serum antioxidants, protective associations with influenza/pneumonia mortality were observed for vitamin C and *α*-tocopherol (online supplementary material, Supplemental Table 7).

## Discussion

The results of this prospective population study suggest that antioxidants status may reduce mortality risk from influenza and pneumonia in US adults. The associations between serum antioxidants status and influenza/pneumonia morality were confirmed in different statistical models and sensitivity analyses with additional covariates. In particular, the protective association was observed even in our secondary analysis with dietary intake of vitamin C, supporting the importance of vitamin C supplementation on prevention of worsening influenza or pneumonia after viral infection. These findings are of value in that, for the first time in a prospective analysis, the associations of antioxidants on influenza-related mortality were demonstrated.

Vitamins C, A and E and carotenoids (e.g. carotene and lycopene) are well-known antioxidants^(19,31)^. The associations of antioxidants status with influenza/pneumonia mortality observed in the current study can be explained by the antioxidant activity of the micronutrients. Antioxidant activity has been suggested to reduce lung damage and prevent viral mutation^(2,5,7)^. In a study with influenza-infected mice, combined treatments of vitamin C and vitamin E showed protective effects, and their antioxidant potential was suggested as a main mechanism of the effects^(32)^.

Antioxidants can also play beneficial roles against influenza infection by modulating the production of proinflammatory cytokines, e.g. interferons and IL. For example, vitamin E supplementation reduced influenza severity in influenza-infected old mice by increasing the production of T helper 1 cytokines such as IL-2 and interferon-*γ*^(33)^. Mice with vitamin C-insufficient genotype Gulo (−/−) were more susceptible to H3N2 influenza A virus infection than wild-type mice^(34)^. Gulo (−/−) mice also showed increased viral titers as well as decreased production of interferon-*α*/*β* in the lung, and these adverse effects of influenza infection on immune response in Gulo (−/−) mice were ameliorated by vitamin C supplementation^(34)^.

It should be noted that the associations observed in this study do not directly indicate an association between short-term or therapeutic use of the antioxidants and incidence of influenza or pneumonia. This study associated serum concentrations of the antioxidants at baseline with mortality data followed up over 16 years on average. By contrast, public interest is often focused on the preventive effects of short-term intake of antioxidants in high doses during pandemic virus infections or their therapeutic effects after the onset of the infection rather than the effects of long-term daily intake of the antioxidants^(1)^. Therefore, RCT with short-term high doses or therapeutic treatment may be warranted.

It is interesting that the protective associations of serum vitamin C, carotene and TAC were greatest at the third quartiles and weakened at the fourth quartiles (Table 3). These nonlinear relationships were confirmed with continuous serum concentrations of these nutrients (Fig. 1). These observations suggest that the risk of influenza/pneumonia mortality among people with deficient nutrition may be effectively reduced by nutritional supplementation. It is also possible that the highest quartile for each of the antioxidants represents individuals who are voluntarily taking supplements because of co-morbidities that were unaccounted for in our study^(35)^ but may have attenuated any perceived beneficial effect of antioxidants, although additional adjustment for supplement use did not change the HR (online supplementary material, Supplemental Table 2). Another explanation for the U-shaped associations is that toxic substances that are correlated with antioxidant food consumption may counteract beneficial antioxidant effects. For example, consumption of an excess amount of carrot, a source of *β*-carotene, can lead to exposure to toxic substances such as nitrosamines and nitrites^(36)^. Excessive intake of vitamin C can also have adverse effects on kidney, stomach, blood pressure and pancreas^(37)^. Moreover, it is reported that extraordinary high levels of serum carotene can be found among those with hypothyroidism or diabetes^(38)^. In any case, in the US general population, the amount of vitamin C intake from foods and supplements has decreased over time^(29,39)^ with the mean intake of vitamin C from foods in the current US population (estimation from NHANES 2017–2018^(35)^) estimated to be approximately 70 % of that in the NHANES-III (1988–1994) population^(40)^. Considering the higher likely prevalence of vitamin C deficiency expected in the current US population as well as the other known health benefits of vitamin C^(41)^, recommending higher intake of vitamin C-rich foods or dietary supplementation could be a good strategy for lowering population-wide risks of disease severity in future viral epidemics, especially for those with nutritional deficiencies. In addition, given previous epidemiologic and experimental evidence on the protective effects of carotene against influenza or pneumonia^(42,43)^, further studies to confirm the possible role of carotene supplementation on influenza/pneumonia mortality are warranted.

The current study has several limitations. First, the serum concentrations of the antioxidants were measured only once at baseline. The single-measured serum concentrations may not represent the long-term antioxidants status of the participants considering an increasing trend of dietary supplement use with age in the US general population^(44,45)^. To remove possible measurement error caused by the temporal change in antioxidant status, we performed sensitivity analyses by excluding the participants with shorter follow-up or very old adults (≥85 years) (online supplementary material, Supplemental Table 6). The age restriction produced consistent results, but associations of vitamin C and TAC were diminished after restricting follow-up time, although we cannot rule out the influence of limited statistical power due to the reduced number of mortality cases. Further studies with repeated measurements of antioxidant status should provide a clearer picture of this issue.

Second, to prevent participant re-identification, the public-use linked mortality data of NHANES-III were perturbated by introducing random noise to follow-up time and underlying cause of death^(46)^. The misclassification caused by the data perturbation is likely to be nondifferential, which generally leads to bias of the associations towards the null. Therefore, the associations observed in this study may be underestimated.

Third, because information on multiple causes of death was not available, only participants who died from influenza or pneumonia as the primary cause of death could be considered. However, influenza-related deaths are often caused by multiple diseases such as other respiratory or CVD^(47)^. Antioxidant therapy has been suggested to alleviate severe influenza-related complications^(48)^. Therefore, inclusion of mortality from influenza-related complications may produce greater HR compared to the observations in this study.

Fourth, we applied a single imputed age (93 years) to the top-coded age. Due to insufficient information on age, the potential confounding effects of age could not be fully adjusted, which potentially leads to bias. Even after excluding participants with ≥85 years of age, however, we observed robust associations of serum vitamin C, carotene, lycopene and TAC with influenza/pneumonia mortality, suggesting that the potential bias caused by the imputed age was minimal.

## Conclusion

The current study suggests that antioxidant intake measured by serum concentrations may reduce mortality risk from influenza or pneumonia in the US general population. The associations were robust for serum vitamin C, carotene and lycopene suggesting supplementation with these nutrients may be a recommendation for preparing for future virus pandemic. However, the findings of this study do not directly provide evidence of short-term treatment of these antioxidants in high doses for preventive purpose with respect to the COVID-19 outbreak. Therefore, further confirmation of the association between antioxidants use and infectious diseases in other populations in different settings and in different dosing regimens (e.g. shorter-term/high-dose supplementation in association with virus infection) is warranted.

## Supplementary Material

1

2

## Figures and Tables

**Table 1:** Survey-weighted characteristics of the study participants by quartile of serum total antioxidant capacity (*n* 7428)

			Serum total antioxidant capacity (mg VCE/dl)							
Characteristics	Total population (*n* 7428)		Q1 (< 0·8) (*n* 1857)		Q2 (0·8–< 1·2) (*n* 1857)		Q3 (1·2–< 1·6) (*n* 1858)		Q4 (≥1·6) (*n* 1856)	

Influenza/pneumonia deaths (*n*)	154		39		38		33		44	
Follow-up year										
Median	168		14·2		17·8		19·2		16·6	
Q1–Q3	8·5–23·2		7·2–22·5		8·6–23·3		9·4–23·4		8·9–22·8	
Mortality from influenza/pneumonia (per 1000 person-years)	Incident rate	95 % CI	Incident rate	95 % CI	Incident rate	95 % CI	Incident rate	95 % CI	Incident rate	95 % CI
Unweighted	1·32	1·12, 1·55	1·46	1·04, 1·99	1·28	0·90, 1·75	1·07	0·74, 1·51	1·51	1·10, 2·03
Weighted	0·88	0·88, 0·88	1·21	1·20, 1·21	0·96	0·96, 0·96	0·46	0·46, 0·46	1·22	1·22, 1·23
Baseline continuous variables	Mean	95 % CI	Mean	95 % CI	Mean	95 % CI	Mean	95 % CI	Mean	95 % CI
Age (years)	61·4	60·8, 62·1	59·3	58·6, 59·9	60·4	59·5, 61·3	61·1	60·3, 61·9	64·0	62·9, 65·1
BMI (kg/m^2^)	27·3	27·1, 27·5	27·7	27·2, 28·2	28·2	27·8, 28·5	27·3	27·0, 27·7	26·4	26·1, 26·7
Serum total cholesterol (mg/dl)	222	219, 223	213·4	210·3, 216·5	217·1	214·8, 219·5	220·0	216·9, 223·1	232·0	228·5, 235·5
Serum cotinine (mg/l)*	1·09	0·90, 1·33	8·60	7·06, 10·49	1·14	0·94, 1·39	0·57	0·47, 0·69	0·46	0·38, 0·56
Baseline categorical variables	%		%		%		%		%	
Female	53·9		41·3		48·1		52·3		68·7	
Race/ethnicity										
Non-Hispanic White	82·8		76·0		78·0		83·9		90·3	
Non-Hispanic Black	8·2		14·7		10·9		6·3		3·4	
Mexican American	3·0		3·7		3·9		2·9		2·0	
Other	6·0		5·6		7·3		6·9		4·3	
Education										
< High school	33·0		43·8		36·2		28·1		27·6	
High school diploma	48·8		45·8		44·4		50·8		52·4	
> High school	18·2		10·4		19·4		21·1		20·0	
PIR ≥ 10^†^	90·0		82·9		88·5		92·1		94·0	
Smoking status										
Never	42·1		28·4		41·2		45·2		49·3	
Former	36·8		29·2		39·6		40·3		36·6	
Current	21·1		42·3		19·2		14·5		14·2	
Alcohol consumption ≥4 time/month^‡^	33·0		34·4		32·4		33·6		31·9	
Supplement use^§^	46·6		19·3		32·5		48·7		74·7	

* Serum cotinine had 85 missing observations, and geometric means and 95% CI are presented.

† Poverty:income ratio (PIR) had 778 missing observations.

‡ Alcohol consumption had 283 missing observations.

§ Supplement use had 6 missing observations.

**Table 2:** Concentrations of antioxidants and total antioxidant capacity in serum of the study participants (*n* 7428)

	Serum concentration (μg/dl)	
Antioxidant	Median	Q1–Q3

Vitamin C	740	360–1050
Vitamin A (as retinol)	60	51–71
Vitamin E (as *α*-tocopherol)	1185	996–1485
Sum of *α*- and *β*-carotene	22	13–36
*β*-cryptoxanthin	8	5–13
Lutein + zeaxanthin	22	16–31
Lycopene	17	10–25
Total antioxidant capacity^†^	1214	802–1588

† Sum of vitamin C equivalent antioxidant capacity (VCEAC) of serum antioxidants was presented (unit: μg VCE/dl).

**Table 3:** Association of serum antioxidants with mortality from influenza/pneumonia in the study population (*n* 7428)

		Model 1			Model 2		
	Deaths/total	HR	95% CI	*P*	HR	95 % CI	*P*

Vitamin C (mg/dl)							
Q1 (< 0 37)	41/1877	reference			reference		
Q2 (0·37–< 0·75)	36/1892	052	0·27, 1·03	0·06	0·57	0·30, 1·11	0·10
Q3 (0·75–< 1·06)	37/1848	0·34	0·17, 0·71	0·004	0·38	0·19, 0·77	0·007
Q4 (≥1·06)	40/1811	0·48	0·22, 1·04	0·06	0·57	0·27, 1·17	0·13
*P*_for trend_				0·08			0·15
Vitamin A as retinol (μg/dl)							
Q1 (< 51)	40/1982	reference			reference		
Q2 (51–< 61)	34/1907	0·60	0·33, 1·12	0·11	0·58	0·32, 1·06	0·08
Q3 (61–< 72)	44/1753	0·81	0·54, 1·22	0·31	0·75	0·49, 1·16	0·20
Q4 (≥72)	36/1786	0·68	0·38, 1·21	0·19	0·63	0·34, 1·15	0·13
*P*_for trend_				0·37			0·28
Vitamin E as *α*-tocopherol (μg/dl)							
Q1 (< 963)	37/1866	reference			reference		
Q2 (963–< 1186)	37/1852	0·98	0·56, 1·71	0·95	0·95	0·54, 1·67	0·85
Q3 (1186–< 1486)	35/1854	0·84	0·49, 1·46	0·54	0·80	0·44, 1·48	0·49
Q4 (≥1486)	45/1856	1·03	0·63, 1·70	0·89	0·96	0·49, 1·88	0·91
*P*_for trend_				0·93			0·89
Sum of *α*- and *β*-carotene (μg/dl)							
Q1 (< 14)	33/1888	reference			reference		
Q2 (14–< 23)	43/1896	0·75	0·41, 1·38	0·36	0·76	0·41, 1·40	0·37
Q3 (23–< 37)	29/1830	0·28	0·16, 0·49	<0·0001	0·29	0·16, 0·51	<0·0001
Q4 (≥37)	49/1814	0·63	0·38, 1·05	0·08	0·70	0·41, 1·19	0·19
*P*_for trend_				0·04			0·11
*β*-cryptoxanthin (μg/dl)							
Q1 (< 6)	45/2026	reference			reference		
Q2 (6–< 9)	32/1867	0·54	0·28, 1·05	0·07	0·55	0·28, 1·08	0·08
Q3 (9–< 14)	44/1791	0·65	0·34, 1·22	0·18	0·69	0·36, 1·32	0·26
Q4 (≥14)	33/1744	0·58	0·31, 1·08	0·09	0·62	0·32, 1·19	0·15
*P*_for trend_				0·15			0·24
Lutein + zeaxanthin (μg/dl)							
Q1 (< 17)	37/2021	reference			reference		
Q2 (17–< 23)	41/1780	0·98	0·61, 1·59	0·95	1·01	0·61, 1·68	0·97
Q3 (23–< 32)	38/1882	0·77	0·45, 1·30	0·33	0·80	0·45, 1·41	0·44
Q4 (≥32)	38/1745	0·79	0·45, 1·39	0·42	0·82	0·44, 1·53	0·53
*P*_for trend_				0·28			0·40
Lycopene (μg/dl)							
Q1 (< 11)	62/1897	reference			reference		
Q2 (11–< 18)	38/2044	0·60	0·36, 1·01	0·05	0·59	0·35, 1·00	0·05
Q3 (18–< 26)	28/1717	0·50	0·28, 0·90	0·02	0·48	0·27, 0·85	0·01
Q4 (≥26)	26/1770	0·47	0·25, 0·86	0·01	0·43	0·23, 0·83	0·01
*P*_for trend_				0·02			0·01
Total antioxidant capacity (mg VCE/dl)							
Q1 (< 0 8)	39/1857	reference			reference		
Q2 (0·8–< 1·2)	38/1857	0·66	0·33, 1·32	0·24	0·70	0·35, 1·45	0·34
Q3 (1·2–< 1·6)	33/1858	0·27	0·13, 0·55	0·0004	0·30	0·15, 0·59	0·0006
Q4 (≥1·6)	44/1856	0·58	0·28, 1·23	0·16	0·65	0·31, 1·35	0·25
*P*_for trend_				0·13			0·20

Hazard ratios (HR) and 95% CI were estimated using survey-weighted Cox proportional hazards models with attained age as the time scale. Model 1: adjusted for sex, race/ethnicity and NHANES III phase. Model 2: further adjusted for education, cholesterol, BMI and smoking history.
