# Supplementary material for: Serum antioxidant status and mortality from influenza and pneumonia in US adults
Source: Public Health Nutr. 2022 Jan 10;25(12):3466–75. doi: 10.1017/S1368980022000027 (PMC9271125; doi:10.1017/S1368980022000027)
Supplement: Supplementary file 1 [file S1368980022000027sup.zip › S1368980022000027sup001.docx]

**<Supplementary Material>**

**Title: Serum antioxidant status and mortality from influenza and pneumonia in US Adults**

**Table of Contents**

**Supplementary Methods**	*2*

**Supplementary Figure 1** Procedure for selection of the study population from NHANES III population	*3*

**Supplementary Figure 2** Spearman’s correlation coefficients among the serum concentrations and dietary intakes of the antioxidants and total antioxidant capacity (TAC) among the participants having both serum concentrations of antioxidants and dietary intakes of antioxidants	*4*

**Supplementary Table 1** Association of serum antioxidant groups by recommendations with mortality from influenza/pneumonia in the study population	*5*

**Supplementary Table 2** Association of serum antioxidants with mortality from influenza/pneumonia with adjustment for additional potential confounders	*6*

**Supplementary Table 3** Several disease statuses in the study population	*8*

**Supplementary Table 4** Association of serum antioxidants with mortality from influenza/pneumonia with additional adjustment for disease status	*9*

**Supplementary Table 5** Association of serum antioxidants with mortality from influenza/pneumonia with consideration of additional nutrients	*11*

**Supplementary Table 6** Association of serum antioxidants with mortality from influenza/pneumonia in population with restricted follow-up time and/or age at baseline	*13*

**Supplementary Table 7** Association of energy-adjusted dietary intake of antioxidants with mortality from influenza/pneumonia in the study population for dietary antioxidants	*14*

**References**	*15*

**Supplementary Methods**

*Study population for dietary antioxidants*

Population for dietary antioxidants was selected using same exclusion criteria with the main population (population for serum antioxidants), but we included participants with data on dietary intake of antioxidants regardless of missing values in serum levels of the antioxidants (Supplementary Figure 1).

*Dietary antioxidant intake*

NHANES III provides 24 h recall-based daily intake of nutrients calculated using United States Department of Agriculture (USDA) database [Survey nutrient data bases for NHANES III, Phase 1 (1993) and Phase 2 (1995)]^(1)^. Among the nutrients whose daily intake was calculated, we included vitamin C, vitamin A, vitamin E, and β-carotene for the analyses. Daily intake of α-carotene, β-cryptoxanthin, lutein, zeaxanthin, and lycopene were not calculated by the USDA database.

*Statistical analysis for dietary antioxidants*

We used survey-weighted Cox proportional hazard models to associate dietary intake of antioxidants with influenza/pneumonia mortality with same covariates with the main analysis for the serum antioxidants. However, to control for possible confounding by total energy intake, we applied “residual model” Willett et al. (1997) suggested^(2)^. For this adjustment for total energy intake, we used energy-adjusted intake of the nutrients as well as included total energy intake (log-transformed) as a covariate in the models.

**Supplementary Figure 1** Procedure for selection of the study population from NHANES III population.

**Supplementary Figure 2** Spearman’s correlation coefficients among the serum concentrations and dietary intakes of the antioxidants and total antioxidant capacity (TAC) among the participants having both serum concentrations of antioxidants and dietary intakes of antioxidants (n = 6975). When the *P*-value is under 0.05, corresponding correlation coefficient was represented with area and color of a circle.

**Supplementary Table 1** Association of serum antioxidant groups by recommendations with mortality from influenza/pneumonia in the study population (n=7428)

|  |  | Deaths/total | Model 1 | |  | Model 2 | |
| --- | --- | --- | --- | --- | --- | --- | --- |
|  |  |  | HR (95% CI) | *P* |  | HR (95% CI) | *P* |
| **Vitamin C (mg/dL)^a^** | |  |  |  |  |  |  |
|  | Low (<0.4) | 45/2005 | 2.31 (1.28, 4.18) | 0.005 |  | 2.09 (1.16, 3.74) | 0.01 |
|  | Normal (0.4–<1.0) | 63/3291 | (reference) |  |  | (reference) |  |
|  | Saturated (≥1.0) | 46/2132 | 1.07 (0.60, 1.92) | 0.81 |  | 1.14 (0.63, 2.06) | 0.67 |
| **Vitamin A as retinol (μg/dL)^b^** | | |  |  |  |  |  |
|  | Deficient (<35.8) | 5/304 | 1.50 (0.50, 4.53) | 0.47 |  | 1.63 (0.53, 5.04) | 0.40 |
|  | Normal (35.8–85.9) | 131/6502 | (reference) |  |  | (reference) |  |
|  | Excess (>85.9) | 18/622 | 1.45 (0.75, 2.77) | 0.27 |  | 1.42 (0.72, 2.79) | 0.31 |
| **Vitamin E (α–tocopherol; μg/dL)** | | |  |  |  |  |  |
|  | Deficient (<711) | 4/353 | 0.92 (0.47, 1.83) | 0.82 |  | 1.05 (0.51, 2.19) | 0.89 |
|  | Normal (711–1792) | 127/6111 | (reference) |  |  | (reference) |  |
|  | Excess (>1792) | 23/964 | 1.08 (0.61, 1.93) | 0.79 |  | 1.07 (0.58, 1.95) | 0.84 |
| HRs and 95% CIs were estimated using survey-weighted Cox proportional hazards models with attained age as the time scale. Model 1: adjusted for sex, race/ethnicity, and NHANES III phase. Model 2: further adjusted for education, cholesterol, body mass index (BMI), and smoking history.  ^a^Serum vitamin C was categorized based on clinical recommendation^(3)^.  ^b^Serum vitamin A was categorized based on clinical recommendation^(4)^.  ^c^Serum vitamin E was categorized based on clinical recommendation^(4)^. | | | | | | | |

**Supplementary Table 2** Association of serum antioxidants with mortality from influenza/pneumonia with additional adjustment for potential confounders

|  | Primary model^a^  (n = 7428) | |  | + Serum cotinine  (n = 7343)^c^ | |  | + PIR  (n = 6650)^d^ | |  | + Alcohol consumption  (n = 7145)^c^ | |  | + Supplement use  (n = 7422)^d^ | |
| --- | --- | --- | --- | --- | --- | --- | --- | --- | --- | --- | --- | --- | --- | --- |
|  | HR (95% CI) | *P* |  | HR (95% CI) | *P* |  | HR (95% CI) | *P* |  | HR (95% CI) | *P* |  | HR (95% CI) | *P* |
| **Vitamin C (mg/dL)** | | | | | | | | | | | |  |  |  |
| Q1 (<0.37) | (reference) |  |  | (reference) |  |  | (reference) |  |  | (reference) |  |  | (reference) |  |
| Q2 (0.37–<0.75) | 0.57 (0.30, 1.11) | 0.10 |  | 0.67 (0.34, 1.31) | 0.24 |  | 0.45 (0.19, 1.04) | 0.06 |  | 0.51 (0.25, 1.06) | 0.07 |  | 0.57 (0.29, 1.12) | 0.10 |
| Q3 (0.75–<1.06) | 0.38 (0.19, 0.77) | 0.007 |  | 0.41 (0.21, 0.82) | 0.01 |  | 0.35 (0.17, 0.72) | 0.004 |  | 0.38 (0.19, 0.75) | 0.005 |  | 0.38 (0.19, 0.78) | 0.008 |
| Q4 (≥1.06) | 0.57 (0.27, 1.17) | 0.13 |  | 0.64 (0.30, 1.37) | 0.25 |  | 0.60 (0.29, 1.24) | 0.17 |  | 0.57 (0.28, 1.17) | 0.13 |  | 0.56 (0.26, 1.20) | 0.14 |
| **Vitamin A as retinol (μg/dL)** | |  |  |  |  |  |  |  |  |  |  |  |  |  |
| Q1 (<51) | (reference) |  |  | (reference) |  |  | (reference) |  |  | (reference) |  |  | (reference) |  |
| Q2 (51–<61) | 0.58 (0.32, 1.06) | 0.08 |  | 0.53 (0.27, 1.00) | 0.05 |  | 0.56 (0.30, 1.07) | 0.08 |  | 0.58 (0.31, 1.08) | 0.09 |  | 0.58 (0.32, 1.06) | 0.08 |
| Q3 (61–<72) | 0.75 (0.49, 1.16) | 0.20 |  | 0.76 (0.50, 1.17) | 0.21 |  | 0.57 (0.36, 0.93) | 0.02 |  | 0.72 (0.46, 1.13) | 0.15 |  | 0.75 (0.49, 1.17) | 0.21 |
| Q4 (≥72) | 0.63 (0.34, 1.15) | 0.13 |  | 0.62 (0.33, 1.14) | 0.12 |  | 0.59 (0.31, 1.14) | 0.12 |  | 0.63 (0.33, 1.18) | 0.15 |  | 0.63 (0.34, 1.17) | 0.15 |
| **Vitamin E (α–tocopherol; μg/dL)** | |  |  |  |  |  |  |  |  |  |  |  |  |  |
| Q1 (<963) | (reference) |  |  | (reference) |  |  | (reference) |  |  | (reference) |  |  | (reference) |  |
| Q2 (963–<1186) | 0.95 (0.54, 1.67) | 0.85 |  | 0.90 (0.53, 1.55) | 0.71 |  | 0.86 (0.49, 1.54) | 0.62 |  | 0.91 (0.53, 1.56) | 0.73 |  | 0.96 (0.53, 1.75) | 0.90 |
| Q3 (1186–<1486) | 0.80 (0.44, 1.48) | 0.49 |  | 0.86 (0.45, 1.61) | 0.63 |  | 0.79 (0.40, 1.58) | 0.51 |  | 0.82 (0.45, 1.49) | 0.51 |  | 0.83 (0.44, 1.58) | 0.57 |
| Q4 (≥1486) | 0.96 (0.49, 1.88) | 0.91 |  | 0.99 (0.51, 1.95) | 0.98 |  | 1.13 (0.56, 2.28) | 0.72 |  | 0.97 (0.49, 1.92) | 0.92 |  | 1.02 (0.48, 2.20) | 0.95 |
| **Sum of α– and β-carotene (μg/dL)** | |  |  |  |  |  |  |  |  |  |  |  |  |  |
| Q1 (<14) | (reference) |  |  | (reference) |  |  | (reference) |  |  | (reference) |  |  | (reference) |  |
| Q2 (14–<23) | 0.76 (0.41, 1.40) | 0.37 |  | 0.80 (0.44, 1.47) | 0.47 |  | 0.67 (0.34, 1.30) | 0.24 |  | 0.68 (0.37, 1.27) | 0.23 |  | 0.76 (0.41, 1.40) | 0.38 |
| Q3 (23–<37) | 0.29 (0.16, 0.51) | <0.0001 |  | 0.32 (0.19, 0.55) | <0.0001 |  | 0.29 (0.16, 0.52) | <0.0001 |  | 0.28 (0.16, 0.49) | <0.0001 |  | 0.29 (0.16, 0.52) | <0.0001 |
| Q4 (≥37) | 0.70 (0.41, 1.19) | 0.19 |  | 0.73 (0.44, 1.22) | 0.23 |  | 0.66 (0.36, 1.21) | 0.18 |  | 0.68 (0.40, 1.15) | 0.15 |  | 0.71 (0.41, 1.22) | 0.21 |

**Supplementary Table 2** (continued)

|  | Primary model^a^  (n = 7428) | |  | + Serum cotinine  (n = 7343)^c^ | |  | + PIR  (n = 6650)^d^ | |  | + Alcohol consumption  (n = 7145)^c^ | |  | + Supplement use  (n = 7422)^d^ | |  |
| --- | --- | --- | --- | --- | --- | --- | --- | --- | --- | --- | --- | --- | --- | --- | --- |
|  | HR (95% CI) | *P* |  | HR (95% CI) | *P* |  | HR (95% CI) | *P* |  | HR (95% CI) | *P* |  | HR (95% CI) | *P* | |
| **β-cryptoxanthin (μg/dL)** | | | | | | | | | | | |  |  |  | |
| Q1 (<6) | (reference) |  |  | (reference) |  |  | (reference) |  |  | (reference) |  |  | (reference) |  | |
| Q2 (6–<9) | 0.55 (0.28, 1.08) | 0.08 |  | 0.58 (0.30, 1.13) | 0.11 |  | 0.49 (0.25, 0.99) | 0.05 |  | 0.50 (0.26, 0.97) | 0.04 |  | 0.56 (0.28, 1.09) | 0.09 | |
| Q3 (9–<14) | 0.69 (0.36, 1.32) | 0.26 |  | 0.72 (0.37, 1.38) | 0.32 |  | 0.69 (0.33, 1.43) | 0.32 |  | 0.67 (0.36, 1.26) | 0.22 |  | 0.69 (0.36, 1.32) | 0.26 | |
| Q4 (≥14) | 0.62 (0.32, 1.19) | 0.15 |  | 0.68 (0.36, 1.29) | 0.23 |  | 0.55 (0.29, 1.03) | 0.06 |  | 0.55 (0.29, 1.04) | 0.07 |  | 0.62 (0.33, 1.19) | 0.15 | |
| **Lutein + zeaxanthin (μg/dL)** | |  |  |  |  |  |  |  |  |  |  |  |  |  | |
| Q1 (<17) | (reference) |  |  | (reference) |  |  | (reference) |  |  | (reference) |  |  | (reference) |  | |
| Q2 (17–<23) | 1.01 (0.61, 1.68) | 0.97 |  | 1.11 (0.68, 1.82) | 0.68 |  | 1.01 (0.55, 1.86) | 0.96 |  | 1.00 (0.57, 1.76) | 0.99 |  | 1.01 (0.61, 1.67) | 0.98 | |
| Q3 (23–<32) | 0.80 (0.45, 1.41) | 0.44 |  | 0.88 (0.51, 1.50) | 0.64 |  | 0.83 (0.44, 1.56) | 0.56 |  | 0.83 (0.45, 1.53) | 0.55 |  | 0.79 (0.45, 1.41) | 0.43 | |
| Q4 (≥32) | 0.82 (0.44, 1.53) | 0.53 |  | 0.89 (0.46, 1.73) | 0.73 |  | 0.78 (0.38, 1.61) | 0.50 |  | 0.87 (0.44, 1.72) | 0.70 |  | 0.81 (0.43, 1.53) | 0.52 | |
| **Lycopene (μg/dL)** | | | | | | | | | | | |  |  |  | |
| Q1 (<11) | (reference) |  |  | (reference) |  |  | (reference) |  |  | (reference) |  |  | (reference) |  | |
| Q2 (11–<18) | 0.59 (0.35, 1.00) | 0.05 |  | 0.54 (0.30, 0.97) | 0.04 |  | 0.56 (0.32, 0.98) | 0.04 |  | 0.58 (0.34, 1.00) | 0.05 |  | 0.59 (0.35, 1.01) | 0.05 | |
| Q3 (18–<26) | 0.48 (0.27, 0.85) | 0.01 |  | 0.50 (0.29, 0.88) | 0.02 |  | 0.46 (0.22, 0.95) | 0.04 |  | 0.41 (0.22, 0.78) | 0.007 |  | 0.48 (0.27, 0.84) | 0.01 | |
| Q4 (≥26) | 0.43 (0.23, 0.83) | 0.01 |  | 0.44 (0.22, 0.85) | 0.01 |  | 0.49 (0.25, 0.95) | 0.04 |  | 0.41 (0.20, 0.81) | 0.01 |  | 0.43 (0.22, 0.82) | 0.01 | |
| **Total antioxidant capacity (mg VCE/dL)** | | | |  |  |  |  |  |  |  |  |  |  |  | |
| Q1 (<0.8) | (reference) |  |  | (reference) |  |  | (reference) |  |  | (reference) |  |  | (reference) |  | |
| Q2 (0.8–<1.2) | 0.70 (0.35, 1.45) | 0.34 |  | 0.76 (0.37, 1.55) | 0.44 |  | 0.56 (0.24, 1.31) | 0.18 |  | 0.64 (0.30, 1.38) | 0.25 |  | 0.71 (0.34, 1.46) | 0.35 | |
| Q3 (1.2–<1.6) | 0.30 (0.15, 0.59) | 0.0006 |  | 0.34 (0.17, 0.67) | 0.002 |  | 0.27 (0.13, 0.54) | 0.0003 |  | 0.29 (0.14, 0.58) | 0.0006 |  | 0.30 (0.15, 0.61) | 0.0009 | |
| Q4 (≥1.6) | 0.65 (0.31, 1.35) | 0.25 |  | 0.71 (0.33, 1.52) | 0.38 |  | 0.72 (0.35, 1.48) | 0.38 |  | 0.65 (0.31, 1.38) | 0.26 |  | 0.66 (0.29, 1.47) | 0.31 | |
| HRs and 95% CIs were estimated using survey-weighted Cox proportional hazards models with attained age as the time scale.  ^a^Primary model (Model 2 in the text): adjusted for sex, race/ethnicity, NHANES III phase, education, cholesterol, body mass index (BMI), and smoking history.  ^b^adjusted for the covariates in the primary model and log-transformed serum cotinine.  ^c^adjusted for the covariates in the primary model and poverty:income ratio (PIR).  ^d^adjusted for the covariates in the primary model and alcohol consumption.  ^e^adjusted for the covariates in the primary model and supplement use. | | | | | | | | | | | |  |  |  | |

**Supplementary Table 3** Several disease statuses (%) in the study population

| Characteristics | Number of missing | Serum total antioxidant capacity (mg VCE/dL) | | | |
| --- | --- | --- | --- | --- | --- |
|  |  | Q1 (<0.8)  (n = 1857) | Q2 (0.8–<1.2)  (n = 1857) | Q3 (1.2–<1.6)  (n = 1858) | Q4 (≥1.6)  (n = 1856) |
| Hypertension based on blood pressure^a^ | 296 | 29.7 | 29.5 | 26.5 | 32.2 |
| Diagnosis of diabetes status | 7 | 8.9 | 13.8 | 9.0 | 7.7 |
| Diagnosis of heart attack | 80 | 8.2 | 8.4 | 7.5 | 7.0 |
| Diagnosis of chronic lung disease^b^ | 4 | 14.7 | 10.2 | 9.1 | 11.3 |
| Diagnosis of any cancer | 3 | 12.0 | 13.9 | 14.4 | 17.4 |
| ^a^Hypertension was defined as a systolic blood pressure of 140 mm Hg or more, or diastolic blood pressure of 90 or more.  ^b^Diagnosis of chronic bronchitis and/or emphysema. | | | | | |

**Supplementary Table 4** Association of serum antioxidants with mortality from influenza/pneumonia with additional adjustment for disease status

|  | + Hypertension  (n=7132)^a^ | |  | + Diabetes  (n=7421)^b^ | |  | + Heart attack  (n=7348)^c^ | |  | + Chronic lung disease  (n=7424)^d^ | |  | + Cancer  (n=7425) | |
| --- | --- | --- | --- | --- | --- | --- | --- | --- | --- | --- | --- | --- | --- | --- |
|  | HR (95% CI) | *P* |  | HR (95% CI) | *P* |  | HR (95% CI) | *P* |  | HR (95% CI) | *P* |  | HR (95% CI) | *P* |
| **Vitamin C (mg/dL)** | | | | | | | | | | | |  |  |  |
| Q1 (<0.37) | (reference) |  |  | (reference) |  |  | (reference) |  |  | (reference) |  |  | (reference) |  |
| Q2 (0.37–<0.75) | 0.58 (0.30, 1.13) | 0.11 |  | 0.56 (0.29, 1.08) | 0.08 |  | 0.56 (0.29, 1.08) | 0.08 |  | 0.57 (0.30, 1.00) | 0.10 |  | 0.57 (0.30, 1.01) | 0.10 |
| Q3 (0.75–<1.06) | 0.38 (0.19, 0.77) | 0.007 |  | 0.38 (0.19, 0.76) | 0.006 |  | 0.38 (0.19, 0.76) | 0.006 |  | 0.39 (0.19, 0.77) | 0.007 |  | 0.38 (0.19, 0.76) | 0.007 |
| Q4 (≥1.06) | 0.58 (0.28, 1.19) | 0.14 |  | 0.57 (0.28, 1.17) | 0.13 |  | 0.57 (0.28, 1.17) | 0.13 |  | 0.56 (0.27, 1.16) | 0.12 |  | 0.57 (0.28, 1.17) | 0.13 |
| **Vitamin A as retinol (μg/dL)** | |  |  |  |  |  |  |  |  |  |  |  |  |  |
| Q1 (<51) | (reference) |  |  | (reference) |  |  | (reference) |  |  | (reference) |  |  | (reference) |  |
| Q2 (51–<61) | 0.58 (0.31, 1.09) | 0.09 |  | 0.58 (0.32, 1.07) | 0.08 |  | 0.58 (0.31, 1.07) | 0.08 |  | 0.58 (0.32, 1.07) | 0.08 |  | 0.59 (0.32, 1.07) | 0.08 |
| Q3 (61–<72) | 0.75 (0.48, 1.16) | 0.20 |  | 0.76 (0.50, 1.16) | 0.20 |  | 0.75 (0.48, 1.16) | 0.20 |  | 0.75 (0.48, 1.16) | 0.19 |  | 0.76 (0.49, 1.17) | 0.21 |
| Q4 (≥72) | 0.66 (0.35, 1.22) | 0.19 |  | 0.63 (0.35, 1.15) | 0.13 |  | 0.63 (0.34, 1.17) | 0.14 |  | 0.63 (0.34, 1.15) | 0.13 |  | 0.64 (0.35, 1.17) | 0.15 |
| **Vitamin E (α–tocopherol; μg/dL)** | | | | | | | | | | | |  |  |  |
| Q1 (<963) | (reference) |  |  | (reference) |  |  | (reference) |  |  | (reference) |  |  | (reference) |  |
| Q2 (963–<1186) | 0.98 (0.55, 1.74) | 0.94 |  | 0.94 (0.54, 1.66) | 0.84 |  | 0.95 (0.54, 1.66) | 0.85 |  | 0.94 (0.53, 1.66) | 0.83 |  | 0.94 (0.53, 1.66) | 0.83 |
| Q3 (1186–<1486) | 0.82 (0.44, 1.54) | 0.54 |  | 0.80 (0.43, 1.47) | 0.47 |  | 0.80 (0.43, 1.48) | 0.48 |  | 0.80 (0.44, 1.47) | 0.47 |  | 0.80 (0.43, 1.49) | 0.49 |
| Q4 (≥1486) | 0.99 (0.50, 1.98) | 0.98 |  | 0.95 (0.49, 1.84) | 0.88 |  | 0.97 (0.49, 1.90) | 0.92 |  | 0.96 (0.49, 1.86) | 0.90 |  | 0.98 (0.50, 1.92) | 0.94 |
| **Sum of α– and β-carotene (μg/dL)** | | |  |  |  |  |  |  |  |  |  |  |  |  |
| Q1 (<14) | (reference) |  |  | (reference) |  |  | (reference) |  |  | (reference) |  |  | (reference) |  |
| Q2 (14–<23) | 0.78 (0.42, 1.43) | 0.42 |  | 0.76 (0.41, 1.39) | 0.37 |  | 0.76 (0.41, 1.40) | 0.38 |  | 0.75 (0.41, 1.47) | 0.35 |  | 0.76 (0.41, 1.41) | 0.39 |
| Q3 (23–<37) | 0.29 (0.17, 0.52) | <0.0001 |  | 0.28 (0.16, 0.51) | <0.0001 |  | 0.29 (0.16, 0.52) | <0.0001 |  | 0.29 (0.16, 0.51) | <0.0001 |  | 0.29 (0.16, 0.52) | <0.0001 |
| Q4 (≥37) | 0.70 (0.41, 1.20) | 0.20 |  | 0.68 (0.40, 1.18) | 0.17 |  | 0.68 (0.40, 1.17) | 0.17 |  | 0.69 (0.40, 1.19) | 0.18 |  | 0.70 (0.41, 1.20) | 0.20 |

**Supplementary Table 4** (continued)

|  | + Hypertension  (n=7132)^a^ | |  | + Diabetes  (n=7421)^b^ | |  | + Heart attack  (n=7348)^c^ | |  | + Chronic lung disease  (n=7424)^d^ | |  | + Cancer  (n=7425) | |
| --- | --- | --- | --- | --- | --- | --- | --- | --- | --- | --- | --- | --- | --- | --- |
|  | HR (95% CI) | *P* |  | HR (95% CI) | *P* |  | HR (95% CI) | *P* |  | HR (95% CI) | *P* |  | HR (95% CI) | *P* |
| **β-cryptoxanthin (μg/dL)** | | | | | | | | | | | |  |  |  |
| Q1 (<6) | (reference) |  |  | (reference) |  |  | (reference) |  |  | (reference) |  |  | (reference) |  |
| Q2 (6–<9) | 0.57 (0.29, 1.11) | 0.10 |  | 0.55 (0.28, 1.08) | 0.08 |  | 0.56 (0.28, 1.09) | 0.09 |  | 0.55 (0.28, 1.08) | 0.08 |  | 0.56 (0.29, 1.10) | 0.09 |
| Q3 (9–<14) | 0.40 (0.37, 1.34) | 0.28 |  | 0.68 (0.36, 1.31) | 0.25 |  | 0.69 (0.36, 1.33) | 0.26 |  | 0.69 (0.36, 1.33) | 0.27 |  | 0.69 (0.36, 1.32) | 0.26 |
| Q4 (≥14) | 0.64 (0.34, 1.23) | 0.18 |  | 0.62 (0.33, 1.19) | 0.15 |  | 0.62 (0.32, 1.19) | 0.15 |  | 0.62 (0.33, 1.19) | 0.15 |  | 0.62 (0.33, 1.19) | 0.15 |
| **Lutein + zeaxanthin (μg/dL)** | |  |  |  |  |  |  |  |  |  |  |  |  |  |
| Q1 (<17) | (reference) |  |  | (reference) |  |  | (reference) |  |  | (reference) |  |  | (reference) |  |
| Q2 (17–<23) | 1.00 (0.59, 1.69) | 0.99 |  | 1.01 (0.61, 1.67) | 0.96 |  | 1.01 (0.61, 1.69) | 0.96 |  | 1.01 (0.61, 1.68) | 0.97 |  | 1.00 (0.61, 1.65) | 1.00 |
| Q3 (23–<32) | 0.80 (0.45, 1.43) | 0.45 |  | 0.80 (0.45, 1.42) | 0.45 |  | 0.79 (0.45, 1.39) | 0.41 |  | 0.80 (0.45, 1.41) | 0.44 |  | 0.79 (0.45, 1.39) | 0.42 |
| Q4 (≥32) | 0.80 (0.42, 1.53) | 0.51 |  | 0.83 (0.44, 1.55) | 0.55 |  | 0.81 (0.43, 1.52) | 0.50 |  | 0.82 (0.43, 1.54) | 0.53 |  | 0.81 (0.44, 1.52) | 0.52 |
| **Lycopene (μg/dL)** | | | | | | | | | | | |  |  |  |
| Q1 (<11) | (reference) |  |  | (reference) |  |  | (reference) |  |  | (reference) |  |  | (reference) |  |
| Q2 (11–<18) | 0.62 (0.37, 1.04) | 0.07 |  | 0.59 (0.35, 1.00) | 0.05 |  | 0.59 (0.35, 1.00) | 0.05 |  | 0.59 (0.35, 1.01) | 0.05 |  | 0.59 (0.35, 1.00) | 0.05 |
| Q3 (18–<26) | 0.50 (0.28, 0.88) | 0.02 |  | 0.48 (0.27, 0.85) | 0.01 |  | 0.48 (0.27, 0.84) | 0.01 |  | 0.48 (0.27, 0.86) | 0.01 |  | 0.48 (0.27, 0.86) | 0.01 |
| Q4 (≥26) | 0.45 (0.24, 0.85) | 0.01 |  | 0.44 (0.23, 0.84) | 0.01 |  | 0.43 (0.22, 0.81) | 0.01 |  | 0.43 (0.23, 0.83) | 0.01 |  | 0.43 (0.23, 0.82) | 0.01 |
| **Total antioxidant capacity (mg VCE/dL)** | | |  |  |  |  |  |  |  |  |  |  |  |  |
| Q1 (<0.8) | (reference) |  |  | (reference) |  |  | (reference) |  |  | (reference) |  |  | (reference) |  |
| Q2 (0.8–<1.2) | 0.72 (0.35, 1.47) | 0.36 |  | 0.69 (0.33, 1.43) | 0.32 |  | 0.70 (0.34, 1.44) | 0.34 |  | 0.71 (0.34, 1.44) | 0.34 |  | 0.71 (0.35, 1.44) | 0.34 |
| Q3 (1.2–<1.6) | 0.29 (0.15, 0.59) | 0.0006 |  | 0.30 (0.15, 0.59) | 0.0006 |  | 0.29 (0.15, 0.59) | 0.0006 |  | 0.30 (0.15, 0.59) | 0.0006 |  | 0.30 (0.15, 0.59) | 0.0006 |
| Q4 (≥1.6) | 0.67 (0.32, 1.38) | 0.27 |  | 0.65 (0.31, 1.35) | 0.25 |  | 0.64 (0.31, 1.34) | 0.24 |  | 0.65 (0.31, 1.34) | 0.24 |  | 0.65 (0.32, 1.35) | 0.25 |
| HRs and 95% CIs were estimated using survey-weighted Cox proportional hazards models with attained age as the time scale.  Primary model (Model 2 in the text): adjusted for sex, race/ethnicity, NHANES III phase, education, cholesterol, body mass index (BMI), and smoking history.  ^a^adjusted for the covariates in the primary model and hypertension.  ^b^adjusted for the covariates in the primary model and diabetes.  ^c^adjusted for the covariates in the primary model and heart attack.  ^d^adjusted for the covariates in the primary model and chronic lung disease (diagnosis of chronic bronchitis or emphysema).  ^e^adjusted for the covariates in the primary model and cancer. | | | | | | | | | | | | | | |

**Supplementary Table 5** Association of serum antioxidants with mortality from influenza/pneumonia with consideration of additional nutrients (n=7428)

|  | Primary model^a^ | | |  |  |  | + vitamin C^b^ | |  | + lycopene^c^ | |
| --- | --- | --- | --- | --- | --- | --- | --- | --- | --- | --- | --- |
|  | HR (95% CI) | *P* | |  |  |  | HR (95% CI) | *P* |  | HR (95% CI) | *P* |
| **Vitamin C (mg/dL)** | | | | | | | | | | | |
| Q1 (<0.37) | (reference) |  | |  |  |  | (Not applicable) |  |  | (reference) |  |
| Q2 (0.37–<0.75) | 0.52 (0.27, 1.03) | 0.06 | |  |  |  |  |  |  | 0.62 (0.32, 1.20) | 0.15 |
| Q3 (0.75–<1.06) | 0.34 (0.17, 0.71) | 0.004 | |  |  |  |  |  |  | 0.42 (0.21, 0.84) | 0.02 |
| Q4 (≥1.06) | 0.48 (0.22, 1.04) | 0.06 | |  |  |  |  |  |  | 0.62 (0.31, 1.26) | 0.19 |
| **Vitamin A as retinol (μg/dL)** | | |  |  |  |  |  |  |  |  |  |
| Q1 (<51) | (reference) |  | |  |  |  | (reference) |  |  | (reference) |  |
| Q2 (51–<61) | 0.60 (0.33, 1.12) | 0.11 | |  |  |  | 0.60 (0.33, 1.08) | 0.09 |  | 0.62 (0.34, 1.14) | 0.13 |
| Q3 (61–<72) | 0.81 (0.54, 1.22) | 0.31 | |  |  |  | 0.78 (0.50, 1.22) | 0.27 |  | 0.79 (0.51, 1.22) | 0.28 |
| Q4 (≥72) | 0.68 (0.38, 1.21) | 0.19 | |  |  |  | 0.66 (0.37, 1.19) | 0.16 |  | 0.65 (0.36, 1.17) | 0.15 |
| **Vitamin E (α–tocopherol; μg/dL)** | | | | | | | | | | | |
| Q1 (<963) | (reference) |  | |  |  |  | (reference) |  |  | (reference) |  |
| Q2 (963–<1186) | 0.98 (0.56, 1.71) | 0.95 | |  |  |  | 1.07 (0.59, 1.96) | 0.82 |  | 1.10 (0.61, 2.02) | 0.73 |
| Q3 (1186–<1486) | 0.84 (0.49, 1.46) | 0.54 | |  |  |  | 0.98 (0.51, 1.90) | 0.96 |  | 0.95 (0.50, 1.83) | 0.88 |
| Q4 (≥1486) | 1.03 (0.63, 1.70) | 0.89 | |  |  |  | 1.25 (0.63, 2.47) | 0.52 |  | 1.10 (0.57, 2.12) | 0.77 |
| **Sum of α– and β-carotene (μg/dL)** | | | | | | | | | | | |
| Q1 (<14) | (reference) |  | |  |  |  | (reference) |  |  | (reference) |  |
| Q2 (14–<23) | 0.75 (0.41, 1.38) | 0.36 | |  |  |  | 0.85 (0.44, 1.63) | 0.63 |  | 0.86 (0.47, 1.54) | 0.60 |
| Q3 (23–<37) | 0.28 (0.16, 0.49) | <0.0001 | |  |  |  | 0.34 (0.18, 0.63) | 0.0007 |  | 0.35 (0.20, 0.61) | <0.0001 |
| Q4 (≥37) | 0.63 (0.38, 1.05) | 0.08 | |  |  |  | 0.87 (0.47, 1.62) | 0.66 |  | 0.88 (0.52, 1.49) | 0.63 |

**Supplementary Table 5** (continued)

|  | Primary model^a^ | |  |  |  | + vitamin C^b^ | |  | + lycopene^c^ | |
| --- | --- | --- | --- | --- | --- | --- | --- | --- | --- | --- |
|  | HR (95% CI) | *P* |  |  |  | HR (95% CI) | *P* |  | HR (95% CI) | *P* |
| **β-cryptoxanthin (μg/dL)** | | | | | | | | | | |
| Q1 (<6) | (reference) |  |  |  |  | (reference) |  |  | (reference) |  |
| Q2 (6–<9) | 0.54 (0.28, 1.05) | 0.07 |  |  |  | 0.62 (0.32, 1.20) | 0.16 |  | 0.64 (0.33, 1.25) | 0.19 |
| Q3 (9–<14) | 0.65 (0.34, 1.22) | 0.18 |  |  |  | 0.85 (0.41, 1.73) | 0.64 |  | 0.83 (0.44, 1.55) | 0.56 |
| Q4 (≥14) | 0.58 (0.31, 1.08) | 0.09 |  |  |  | 0.77 (0.39, 1.55) | 0.47 |  | 0.76 (0.40, 1.44) | 0.40 |
| **Lutein + zeaxanthin (μg/dL)** | | | | | | | | | | |
| Q1 (<17) | (reference) |  |  |  |  | (reference) |  |  | (reference) |  |
| Q2 (17–<23) | 0.98 (0.61, 1.59) | 0.95 |  |  |  | 1.06 (0.63, 1.81) | 0.82 |  | 1.12 (0.66, 1.90) | 0.68 |
| Q3 (23–<32) | 0.77 (0.45, 1.30) | 0.33 |  |  |  | 0.89 (0.50, 1.58) | 0.69 |  | 0.93 (0.51, 1.67) | 0.80 |
| Q4 (≥32) | 0.79 (0.45, 1.39) | 0.42 |  |  |  | 0.95 (0.49, 1.81) | 0.87 |  | 1.00 (0.50, 2.02) | 0.99 |
| **Lycopene (μg/dL)** | | | | | | | | | | |
| Q1 (<11) | (reference) |  |  |  |  | (reference) |  |  | (Not applicable) |  |
| Q2 (11–<18) | 0.60 (0.36, 1.01) | 0.05 |  |  |  | 0.61 (0.36, 1.04) | 0.07 |  |  |  |
| Q3 (18–<26) | 0.50 (0.28, 0.90) | 0.02 |  |  |  | 0.51 (0.29, 0.90) | 0.02 |  |  |  |
| Q4 (≥26) | 0.47 (0.25, 0.86) | 0.01 |  |  |  | 0.46 (0.24, 0.87) | 0.02 |  |  |  |
| HRs and 95% CIs were estimated using survey-weighted Cox proportional hazards models with attained age as the time scale.  ^a^Primary model (Model 2 in the text): adjusted for sex, race/ethnicity, NHANES III phase, education, cholesterol, body mass index (BMI), and smoking history.  ^b^Adjusted for the covariates in the primary model and serum vitamin C.  ^c^Adjusted for the covariates in the primary model and serum lycopene. | | | | | | | | | | |

**Supplementary Table 6** Association of serum antioxidants with mortality from influenza/pneumonia in population with restricted follow-up time and/or age at baseline

|  |  | Follow-up < 10 y  (n=2216) | | |  | Age at baseline < 85 y  (n=7062) | | |  | Follow-up < 10 y & age at baseline < 85 y  (n=1910) | | |
| --- | --- | --- | --- | --- | --- | --- | --- | --- | --- | --- | --- | --- |
|  |  | Deaths/total | HR (95% CI) | *P* |  | Deaths/total | HR (95% CI) | *P* |  | Deaths/total | HR (95% CI) | *P* |
| **Vitamin C (mg/dL)** | |  |  |  |  |  |  |  |  |  |  |  |
|  | Q1 (<0.37) | 16/658 | (reference) |  |  | 36/1815 | (reference) |  |  | 12/608 | (reference) |  |
|  | Q2 (0.37–<0.75) | 20/560 | 0.98 (0.40, 2.42) | 0.97 |  | 26/1806 | 0.53 (0.26, 1.07) | 0.08 |  | 11/488 | 1.06 (0.30, 3.71) | 0.93 |
|  | Q3 (0.75–<1.06) | 20/487 | 0.75 (0.29, 1.94) | 0.56 |  | 32/1761 | 0.35 (0.17, 0.72) | 0.005 |  | 16/407 | 0.98 (0.29, 3.30) | 0.97 |
|  | Q4 (≥1.06) | 19/511 | 0.76 (0.22, 2.61) | 0.66 |  | 34/1680 | 0.63 (0.29, 1.33) | 0.22 |  | 14/407 | 1.29 (0.33, 5.03) | 0.71 |
| **Sum of α– and β-carotene (μg/dL)** | | | | |  |  |  |  |  |  |  |  |
|  | Q1 (<11) | 17/595 | (reference) |  |  | 29/1830 | (reference) |  |  | 13/550 | (reference) |  |
|  | Q2 (11–<19) | 21/573 | 0.85 (0.44, 1.63) | 0.63 |  | 38/1816 | 0.78 (0.40, 1.52) | 0.46 |  | 16/503 | 0.54 (0.20, 1.48) | 0.23 |
|  | Q3 (19–<30) | 15/503 | 0.34 (0.18, 0.63) | 0.0007 |  | 22/1733 | 0.25 (0.13, 0.48) | <0.0001 |  | 12/426 | 0.46 (0.15, 1.40) | 0.17 |
|  | Q4 (≥30) | 22/545 | 0.87 (0.47, 1.62) | 0.66 |  | 39/1683 | 0.66 (0.38, 1.17) | 0.16 |  | 12/431 | 0.65 (0.23, 1.82) | 0.41 |
| **Lycopene (μg/dL)** | |  |  |  |  |  |  |  |  |  |  |  |
|  | Q1 (<11) | 36/830 | (reference) |  |  | 49/1723 | (reference) |  |  | 26/683 | (reference) |  |
|  | Q2 (11–<18) | 21/647 | 0.63 (0.28, 1.39) | 0.25 |  | 30/1942 | 0.52 (0.29, 0.92) | 0.02 |  | 13/563 | 0.37 (0.13, 1.05) | 0.06 |
|  | Q3 (18–<26) | 8/417 | 0.23 (0.10, 0.51) | 0.0003 |  | 25/1663 | 0.49 (0.26, 0.92) | 0.03 |  | 6/373 | 0.16 (0.05, 0.49) | 0.001 |
|  | Q4 (≥26) | 10/322 | 0.48 (0.21, 1.10) | 0.08 |  | 24/1734 | 0.43 (0.21, 0.88) | 0.02 |  | 8/291 | 0.39 (0.15, 1.10) | 0.06 |
| **Total antioxidant capacity (mg VCE/dL)** | | | |  |  |  |  |  |  |  |  |  |
|  | Q1 (<0.8) | 15/655 | (reference) |  |  | 33/1794 | (reference) |  |  | 11/607 | (reference) |  |
|  | Q2 (0.8–<1.2) | 21/534 | 1.42 (0.55, 3.69) | 0.47 |  | 29/1768 | 0.64 (0.30, 1.37) | 0.25 |  | 12/455 | 1.35 (0.39, 4.68) | 0.64 |
|  | Q3 (1.2–<1.6) | 20/497 | 0.95 (0.40, 2.24) | 0.91 |  | 29/1774 | 0.25 (0.12, 0.53) | 0.0003 |  | 17/424 | 1.19 (0.36, 3.94) | 0.78 |
|  | Q4 (≥1.6) | 19/530 | 1.03 (0.28, 3.77) | 0.97 |  | 37/1726 | 0.71 (0.33, 1.53) | 0.38 |  | 13/424 | 1.49 (0.33, 6.76) | 0.60 |
| HRs and 95% CIs were estimated using survey-weighted Cox proportional hazards models with attained age as the time scale.  The HR was adjusted for sex, race/ethnicity, NHANES III phase, education, cholesterol, body mass index (BMI), and smoking history. | | | | | | | | | | | | |

**Supplementary Table 7** Association of energy-adjusted dietary intake of antioxidants with mortality from influenza/pneumonia in the study population for dietary antioxidants (n=7228)

|  |  | Deaths/total | Model 1 | |  | Model 2 | |
| --- | --- | --- | --- | --- | --- | --- | --- |
|  |  |  | HR (95% CI) | *P* |  | HR (95% CI) | *P* |
| **Vitamin C (mg/day)** | |  |  |  |  |  |  |
|  | Q1 (<30.5) | 38/1807 | (reference) |  |  | (reference) |  |
|  | Q2 (30.5–<66.5) | 37/1807 | 0.64 (0.34, 1.19) | 0.16 |  | 0.64 (0.36, 1.16) | 0.14 |
|  | Q3 (66.5–<123.0) | 35/1807 | 0.58 (0.27, 1.21) | 0.14 |  | 0.65 (0.33, 1.28) | 0.21 |
|  | Q4 (≥123.0) | 35/1807 | 0.48 (0.28, 0.81) | 0.006 |  | 0.55 (0.34, 0.88) | 0.01 |
|  | *P-*for-trend |  |  | 0.02 |  |  | 0.04 |
| **Vitamin A as retinol (μg/day)** | | |  |  |  |  |  |
|  | Q1 (<142) | 32/1807 | (reference) |  |  | (reference) |  |
|  | Q2 (142–<267) | 36/1807 | 1.13 (0.58, 2.23) | 0.72 |  | 1.14 (0.57, 2.29) | 0.70 |
|  | Q3 (267–<510) | 34/1807 | 0.82 (0.44, 1.53) | 0.54 |  | 0.88 (0.46, 1.68) | 0.70 |
|  | Q4 (≥510) | 43/1807 | 0.58 (0.33, 1.02) | 0.06 |  | 0.64 (0.38, 1.08) | 0.10 |
|  | *P-*for-trend |  |  | 0.02 |  |  | 0.04 |
| **α–tocopherol (mg/day)** | | | | | |  |  |
|  | Q1 (<3.88) | 43/1807 | (reference) |  |  | (reference) |  |
|  | Q2 (3.88–<5.23) | 41/1807 | 0.79 (0.43, 1.48) | 0.46 |  | 0.86 (0.46, 1.60) | 0.63 |
|  | Q3 (5.23–<7.06) | 36/1807 | 0.75 (0.40, 1.39) | 0.36 |  | 0.82 (0.45, 1.50) | 0.52 |
|  | Q4 (≥7.06) | 25/1807 | 0.37 (0.19, 0.71) | 0.003 |  | 0.41 (0.22, 0.78) | 0.007 |
|  | *P-*for-trend |  |  | 0.004 |  |  | 0.008 |
| **γ–tocopherol (mg/day)** | | |  |  |  |  |  |
|  | Q1 (<6.22) | 43/1807 | (reference) |  |  | (reference) |  |
|  | Q2 (6.22–<10.7) | 32/1807 | 0.70 (0.40, 1.20) | 0.20 |  | 0.67 (0.38, 1.20) | 0.18 |
|  | Q3 (10.7–<16.43) | 40/1807 | 1.02 (0.56, 1.86) | 0.95 |  | 1.04 (0.56, 1.94) | 0.90 |
|  | Q4 (≥16.43) | 30/1807 | 0.54 (0.26, 1.13) | 0.10 |  | 0.55 (0.27, 1.12) | 0.10 |
|  | *P-*for-trend |  |  | 0.25 |  |  | 0.28 |
| **β-carotene (μg/day)** | |  |  |  |  |  |  |
|  | Q1 (<602) | 35/1807 | (reference) |  |  | (reference) |  |
|  | Q2 (602–<1274) | 37/1807 | 0.76 (0.36, 1.60) | 0.47 |  | 0.82 (0.38, 1.77) | 0.61 |
|  | Q3 (1274–<3319) | 37/1807 | 0.81 (0.39, 1.67) | 0.57 |  | 0.98 (0.44, 2.16) | 0.95 |
|  | Q4 (≥3319) | 36/1807 | 0.97 (0.51, 1.86) | 0.94 |  | 1.12 (0.54, 2.34) | 0.75 |
|  | *P-*for-trend |  |  | 0.93 |  |  | 0.61 |
| HRs and 95% CIs were estimated using survey-weighted Cox proportional hazards models with attained age as the time scale. Dietary intake of antioxidants was adjusted for total energy intake before the categorization. Model 1: adjusted for sex, race/ethnicity, NHANES III phase, and total energy intake. Model 2: further adjusted for education, cholesterol, body mass index (BMI), and smoking history. | | | | | | | |

**References**

1. CDC/NCHS (Centers for Disease Control and Prevention/National Center for Health Statistics) (1998) The Third National Health and Nutrition Examination Survey (NHANESIII), NHANES III Total Nutrient Intakes File Documentation Series 11, No. 2A (April 1998). https://wwwn.cdc.gov/nchs/nhanes/nhanes3/DataFiles.aspx (accessed June 2021).

2. Willett WC, Howe GR, Kushi LH (1997) Adjustment for total energy intake in epidemiologic studies. *Am J Clin Nutr* **65**, Suppl.,1220S-1228S.

3. Loria CM, Whelton PK, Caulfield LE et al. (1998) Agreement among indicators of vitamin C status. *Am J Epidemiol* **147**, 587-596.

4. Sikkens ECM, Cahen DL, Koch AD et al. (2013) The prevalence of fat-soluble vitamin deficiencies and a decreased bone mass in patients with chronic pancreatitis. *Pancreatology* **13**, 238-242.
